# Ferroptosis in chemotherapy resistance and resensitization in breast cancer: a systematic review of preclinical evidence and translational implications

**DOI:** 10.3389/fonc.2026.1854602

**Published:** 2026-07-01

**Authors:** Kezhen Shen, Heng Zhang, Lei Cai

**Affiliations:** 1 Department of Breast and Thyroid Surgery, Third Xiangya Hospital, Central South University, Changsha, Hunan, China; 2 Fujian Medical University, Fuzhou, Fujian, China

**Keywords:** breast cancer, chemoresistance, ferroptosis, iron metabolism, preclinical evidence

## Abstract

**Background:**

Chemotherapy resistance remains a major obstacle in breast cancer treatment, particularly in triple-negative breast cancer (TNBC). Ferroptosis, an iron-dependent form of regulated cell death, has emerged as a potential therapeutic vulnerability in drug-resistant cancers, but preclinical evidence lacks systematic synthesis.

**Methods:**

PubMed, Scopus, Embase and Web of Science were systematically searched for original studies published between January 2016 and May 2026 investigating ferroptosis in chemotherapy-resistant breast cancer models. Study characteristics, mechanistic data, and outcomes were extracted. Methodological quality was assessed using SYRCLE (*in vivo*) and adapted OHAT (*in vitro*) tools. A narrative synthesis and evidence strength ranking system (Levels A-C) were developed based on study number, *in vivo* validation, and drug class coverage.

**Results:**

Forty−four studies met inclusion criteria, with the majority published since 2024. TNBC was the focus of 47.7% of studies. Mechanisms were classified into six categories: GPX4 axis (28 studies, 63.6%), iron metabolism regulation (8 studies, 18.2%), SLC7A11/xCT pathway (5 studies, 11.4%), and three single−study mechanisms (6.8% combined). GPX4 modulation demonstrated efficacy across all drug classes. Evidence ranking classified the GPX4 axis, iron metabolism regulation, and the SLC7A11/xCT pathway as Level A (strong evidence), and the remaining mechanisms as Level C. Methodological quality was adequate: 88.6% performed rescue experiments, 79.5% included *in vivo* validation. However, reporting of randomization (11.4%) and blinding (0%) in animal studies requires improvement.

**Conclusion:**

Ferroptosis regulation represents a mechanistically diverse and promising strategy to overcome breast cancer chemoresistance. The GPX4 axis, iron metabolism regulation, and the SLC7A11/xCT pathway are the most consistently supported mechanisms, providing a framework for prioritizing translational targets.

**Systematic Review Registration:**

https://www.crd.york.ac.uk/PROSPERO/, identifier CRD420261334827.

## 1 Introduction

Breast cancer remains the most frequently diagnosed malignancy and the leading cause of cancer-related mortality among women worldwide, with an estimated 2.3 million new cases and 685, 000 deaths annually (1). Based on the expression status of estrogen receptor (ER), progesterone receptor (PR), and human epidermal growth factor receptor 2 (HER2), breast cancer is classified into distinct molecular subtypes: luminal A, luminal B, HER2-enriched, and triple-negative breast cancer (TNBC) (2). TNBC, defined by the absence of all three receptors, accounts for approximately 15-20% of all breast cancers and represents the most aggressive subtype, characterized by high proliferative indices, early relapse, and a propensity for visceral metastasis (3, 4).

Due to the lack of targetable hormone receptors and HER2 amplification, patients with TNBC do not benefit from endocrine therapy or HER2-targeted agents. Consequently, cytotoxic chemotherapy remains the mainstay of systemic treatment for both early and advanced TNBC (5). Anthracyclines (e.g., doxorubicin, epirubicin), taxanes (e.g., paclitaxel, docetaxel), and platinum-based agents (e.g., cisplatin, carboplatin) constitute the backbone of TNBC chemotherapy regimens (6). While a subset of patients achieves favorable initial responses, the emergence of intrinsic or acquired chemoresistance occurs in approximately 30-50% of cases, leading to treatment failure, disease progression, and poor long-term survival (7, 8). The mechanisms underlying chemoresistance are multifactorial, encompassing enhanced drug efflux via ATP-binding cassette (ABC) transporters, activation of DNA repair pathways, evasion of apoptosis, and metabolic reprogramming (9, 10). Despite extensive investigation, effective strategies to overcome chemoresistance remain a critical unmet clinical need.

Ferroptosis is a recently characterized form of regulated cell death distinct from apoptosis, necrosis, and autophagy (11). Morphologically, ferroptosis is defined by mitochondrial shrinkage, increased mitochondrial membrane density, and reduction or disappearance of mitochondrial cristae (12). Biochemically, ferroptosis is driven by iron-dependent accumulation of lipid hydroperoxides to lethal levels, a process governed by three core cellular systems: iron metabolism, lipid metabolism, and the glutathione (GSH)-glutathione peroxidase 4 (GPX4) antioxidant axis (13, 14). GPX4 serves as the central negative regulator of ferroptosis by catalyzing the reduction of lipid hydroperoxides to non-toxic lipid alcohols, thereby preventing oxidative damage to cellular membranes (15). The cystine/glutamate antiporter system Xc^–^, composed of SLC7A11 and SLC3A2, supports GPX4 function by supplying cysteine for GSH biosynthesis (16). Iron availability, regulated by transferrin receptor 1 (TFR1), ferritin heavy chain 1 (FTH1), and ferroportin (FPN), determines the extent of Fenton reaction-mediated lipid peroxidation (17). Disruption of any of these regulatory nodes can trigger ferroptosis, making it a potentially exploitable vulnerability in cancer therapy.

Ferroptosis has been increasingly recognized as a key mediator of response to various anticancer therapies, including chemotherapy, radiotherapy, and immunotherapy (18, 19). Notably, several conventional chemotherapeutic agents—such as doxorubicin, paclitaxel, and cisplatin—induce ferroptosis as part of their cytotoxic mechanism (20–22). Furthermore, cancer cells that have acquired resistance to apoptosis often exhibit heightened sensitivity to ferroptosis, suggesting that induction of ferroptosis may represent an effective strategy to circumvent chemoresistance (23, 24). This concept is particularly relevant to TNBC, given its iron- and lipid-rich phenotype, which may confer intrinsic susceptibility to ferroptosis (25, 26). Recent studies further demonstrate that TNBC cells are preferentially vulnerable to ferroptosis-inducing agents compared to other breast cancer subtypes (27).

The past five years have witnessed an exponential increase in preclinical studies investigating the role of ferroptosis in chemotherapy resistance and resensitization in breast cancer. Employing diverse experimental models, genetic and pharmacological interventions, and outcome measures, these studies have generated a rich yet heterogeneous body of evidence. However, this rapidly expanding literature lacks systematic synthesis and critical appraisal. Key questions remain unanswered: What are the predominant ferroptosis regulatory mechanisms exploited to overcome chemoresistance? How do these mechanisms distribute across different chemotherapeutic drug classes? Which mechanisms are supported by the most consistent evidence and thus hold the greatest translational potential? Addressing these questions is essential for prioritizing targets for drug development and designing rational combination strategies for clinical evaluation.

To fill this gap, we conducted a systematic review of preclinical studies investigating ferroptosis−mediated chemoresistance and resensitization in breast cancer. We asked whether diverse ferroptosis regulatory mechanisms converge on a common adaptive vulnerability, and if so, which pathways are most consistently supported by current evidence. Following PRISMA 2020 guidelines, we synthesized evidence from 44 studies published between 2019 and 2026. We characterized the included studies, classified ferroptosis regulatory mechanisms into six categories, and developed an evidence strength ranking system to prioritize targets for future clinical development. Importantly, while the preclinical evidence is promising, it remains preliminary; successful translation will require rigorous validation, improved pharmacological tools, and predictive biomarkers. This systematic review provides a foundational synthesis to guide future research and clinical translation of ferroptosis−targeting strategies for chemotherapy−resistant breast cancer.

## 2 Methods

### 2.1 Search strategy

We systematically searched PubMed, Scopus, Embase, and Web of Science Core Collection for studies published between January 2016 and May 2026. The search strategy was developed *a priori* and combined controlled vocabulary terms (MeSH/Emtree) and free-text keywords applied to titles and abstracts. Boolean operators were used to integrate concepts related to breast cancer, ferroptosis, chemotherapy, and drug resistance. The complete search strategies for all databases are provided in **Supplementary File 1**.

**Figure 1:**
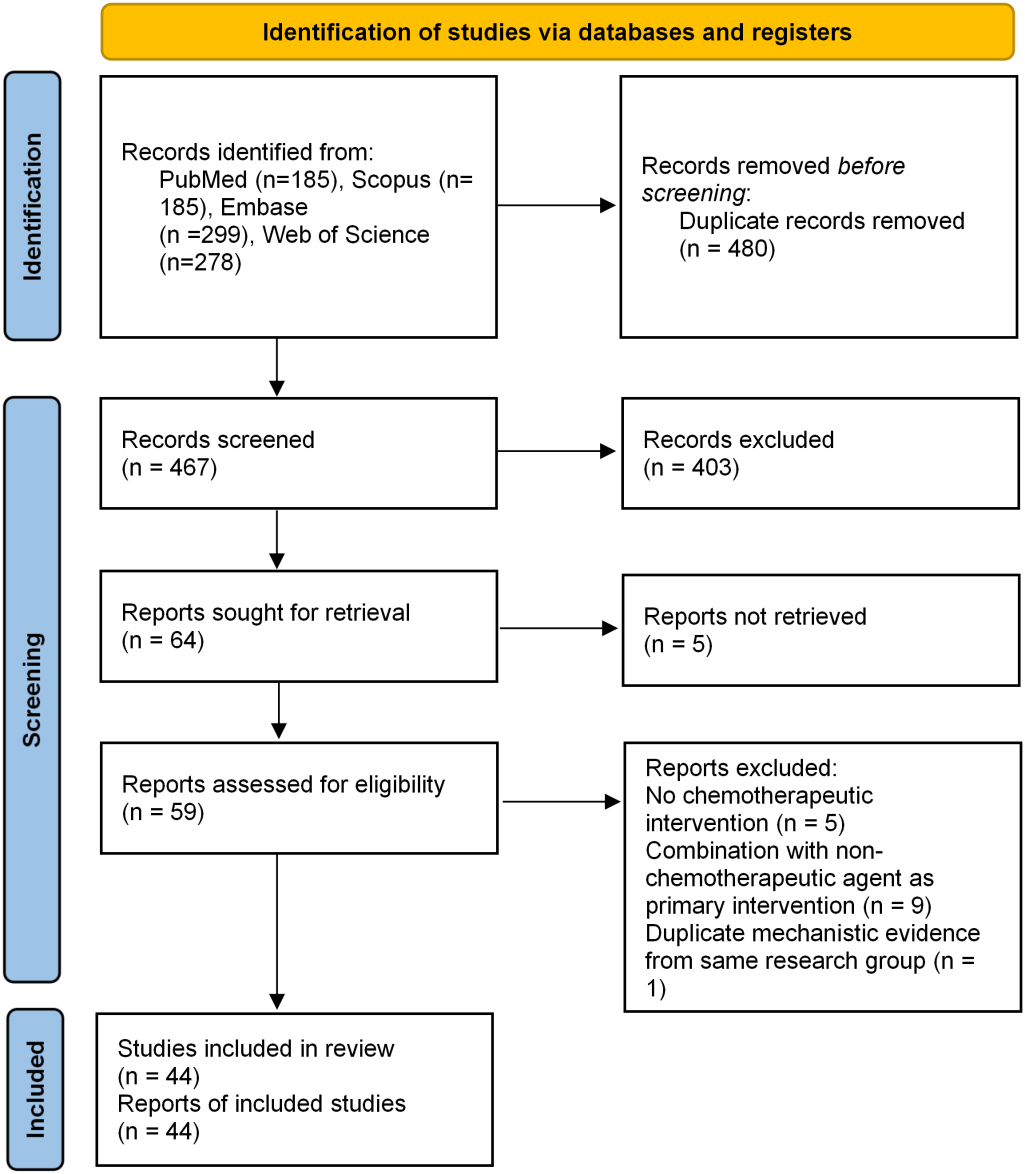
PRISMA flow diagram of study selection process.

The initial search identified 166 records from PubMed, 185 from Scopus, 299 from Embase, and 278 from Web of Science. After removal of duplicate entries, 467 studies remained for title and abstract screening. Sixty-four reports were sought for retrieval at the full-text stage. Of these, five reports were not retrieved due to inaccessible full text. The remaining 59 reports were assessed in detail, of which 15 were excluded for the following reasons: no chemotherapeutic intervention (n = 5); combination with non-chemotherapeutic agent as primary intervention (n = 9); duplicate mechanistic evidence from the same research group (n = 1). Consequently, 44 studies met the eligibility criteria and were included in the final synthesis (**Figure 1**). The study selection process followed the 2020 PRISMA statement (28).

### 2.2 Inclusion and exclusion criteria

#### 2.2.1 Inclusion criteria

Eligible studies were original research articles published in English within the predefined time frame. Studies were required to investigate ferroptosis in the context of chemotherapy response or resistance in breast cancer models. Both *in vitro* and *in vivo* experimental studies were considered, provided that they included breast cancer–specific data.

To be included, investigations had to evaluate at least one conventional cytotoxic chemotherapeutic agent commonly used in breast cancer treatment, such as doxorubicin, paclitaxel, cisplatin, or cyclophosphamide. Studies were expected to incorporate a resistance-relevant framework, including established drug-resistant cell lines, comparative sensitivity analyses between resistant and parental models, or measurable alterations in drug responsiveness. Furthermore, eligible studies were required to demonstrate functional involvement of ferroptosis in modulating chemotherapy response. This included genetic or pharmacologic manipulation of ferroptosis regulators, assessment of lipid peroxidation or iron-dependent cell death markers, and quantitative evaluation of treatment outcomes such as half maximal inhibitory concentration, cell viability, apoptosis assays, clonogenic survival, or tumor growth inhibition. Only full-length peer-reviewed research articles were considered.

#### 2.2.2 Exclusion criteria

Studies were excluded if they did not specifically address breast cancer or lacked chemotherapy-related experimental context. Articles focusing solely on ferroptosis biology without evaluating chemotherapy response were not eligible. Research investigating endocrine therapy, targeted therapy, immunotherapy, or poly(ADP-ribose) polymerase inhibitors without incorporation of conventional cytotoxic chemotherapy was excluded. Review articles, systematic reviews, meta-analyses, conference abstracts, editorials, letters, case reports, protocols, and non–peer-reviewed publications were not considered. Bioinformatics-only studies lacking experimental validation, computational modeling without functional assays, and studies without quantitative outcome measures were excluded. Articles without accessible full text or published in languages other than English were also omitted from the analysis.

### 2.3 Study selection process

All records identified through database searching were screened in two stages. Titles and abstracts were first reviewed to remove clearly irrelevant articles. Full-text evaluation was subsequently performed for potentially eligible studies according to the predefined inclusion and exclusion criteria. Two reviewers independently conducted the screening process. Disagreements were resolved through discussion until consensus was reached. Reasons for exclusion at the full-text stage were documented using predefined exclusion codes to ensure transparency. The final selection of studies included in qualitative synthesis was summarized using a PRISMA flow diagram. The completed PRISMA 2020 checklist is provided as **Supplementary File 2**.

### 2.4 Data extraction

Data were extracted using a predefined standardized template. The following information was collected from each study: first author, publication year, breast cancer subtype, chemotherapeutic agent, experimental system (cell lines, xenograft models, or other *in vivo* platforms), resistance model type (intrinsic or acquired), ferroptosis-related targets or pathways investigated, intervention strategy, comparator groups, and key quantitative outcomes. Additional methodological details, including the presence of control groups, implementation of ferroptosis rescue experiments, number of biological replicates, and statistical reporting methods, were recorded when available. Data extraction was performed by one reviewer and independently verified by a second reviewer to minimize errors and ensure consistency.

### 2.5 Methodological quality assessment

Given the preclinical nature of the included studies, methodological rigor was assessed using two complementary tools tailored for mechanistic oncology research. Two reviewers independently assessed each study, with disagreements resolved through consensus.

For *in vivo* experiments, SYRCLE’s Risk of Bias tool was applied, evaluating eight domains: sequence generation, baseline characteristics, allocation concealment, random housing, blinding, incomplete outcome data, selective outcome reporting, and other sources of bias. Each domain was scored as 1 (low risk) or 0 (high risk/unclear), resulting in a total score ranging from 0 to 8. Studies were then categorized as having low (7-8), moderate (4-6), or high (0-3) risk of bias (29).

For *in vitro* experiments, a quality assessment tool adapted from the OHAT (Office of Health Assessment and Translation) framework was used. This tool assessed seven key domains: intervention description, outcome measurement, statistical analysis, control group setup, data integrity (≥3 independent replicates), selection bias (use of authenticated cell lines/passage control), and outcome consistency (use of ≥2 complementary assays). Each domain was scored 1 (adequately reported) or 0 (inadequately reported/unclear), with a maximum score of 7. Studies scoring 6–7 were considered low risk, 4–5 moderate risk, and 0–3 high risk (30).

### 2.6 Data synthesis

Due to substantial heterogeneity in chemotherapeutic agents, experimental platforms, resistance models, ferroptosis regulators, and outcome measurements, quantitative meta-analysis was not performed. Instead, a structured narrative synthesis was conducted. Studies were grouped according to chemotherapeutic class and mechanistic categories, including canonical antioxidant pathways, iron metabolism regulation, lipid peroxidation modulation, and tumor microenvironment–associated mechanisms. Comparative analysis focused on the consistency of findings, reproducibility across independent models, and integration of *in vitro* and *in vivo* evidence. This approach allowed systematic evaluation of emerging mechanistic patterns while accounting for methodological diversity.

To further integrate quantitative, methodological, and translational dimensions, we developed an evidence strength ranking system (Levels A–C) based on three criteria: number of studies, proportion with *in vivo* validation, and breadth of drug classes covered. The thresholds were selected as follows: ≥5 studies to avoid single−study driven classification (Level A), ≥50% *in vivo* validation as a pragmatic cutoff for translational relevance, and ≥2 drug classes to ensure cross−drug generalizability. These criteria are exploratory and informed by best practices in preclinical evidence synthesis, drawing conceptual inspiration from frameworks such as the Grading of Recommendations Assessment, Development and Evaluation (GRADE). The proposed ranking system was designed as an exploratory prioritization framework rather than a definitive evidence hierarchy. Detailed application of this ranking to the included mechanisms is presented in Section 3.5 and **Table 1**.

**Table 1:** Evidence strength ranking of ferroptosis mechanisms.

Mechanism	Total studies (n)	High robustness (n, %)	With *in vivo* validation (n, %)	Drug classes covered	Evidence strength level
GPX4 axis	28	26 (92.9%)	27 (96.4%)	Taxane + Other + Anthracycline + Platinum (≥3 classes)	Level A (Strong evidence)
Iron metabolism regulation	8	6 (75%)	4 (50%)	Anthracycline + Platinum + Taxane (≥3 classes)	Level A (Strong evidence)
SLC7A11/xCT pathway	5	5 (100%)	4 (80%)	Anthracycline + Platinum + Taxane (≥3 classes)	Level A (Strong evidence)
Lipid peroxidation regulation	1	1 (100%)	1 (100%)	Anthracycline (single)	Level C (Preliminary evidence)
Non-canonical/Other	1	1 (100%)	1 (100%)	Taxane (single)	Level C (Preliminary evidence)
Tumor microenvironment related	1	1 (100%)	1 (100%)	Anthracycline (single)	Level C (Preliminary evidence)

Level A (Strong evidence): ≥5 studies AND ≥50% in vivo validation AND ≥2 drug classes; Level B (Moderate evidence): 3–4 studies OR in vivo validation 50%; Level C (Preliminary evidence): ≤2 studies OR single drug class only. The “High Robustness” classification was calculated based on five indicators: lipid peroxidation measurement, iron assessment, rescue experiment, in vivo validation, and statistical methods. Each indicator was scored 1 (present/adequately reported) or 0 (absent/unclear), yielding a total robustness score ranging from 0 to 5. Studies scoring ≥4 were classified as High Robustness, 2–3 as Moderate Robustness, and ≤1 as Limited Robustness. Detailed scoring for each study is provided in **Supplementary Table S1**.

## 3 Results

### 3.1 Study characteristics

A total of 44 preclinical studies (31–74) published between 2019 and 2026 met the eligibility criteria and were included in this systematic review (**Figure 1**, PRISMA flow diagram). The annual distribution of publications showed a marked increase in recent years, with the majority of studies published between 2024 and 2026 (**Supplementary Figure 1**), indicating that ferroptosis−mediated chemoresistance is an emerging research hotspot.

Regarding breast cancer subtypes, 21 studies (47.7%) exclusively focused on triple−negative breast cancer (TNBC), 18 studies (40.9%) used mixed subtypes (including TNBC, HER2+, and luminal models), four studies (9.1%) focused on luminal or HER2−negative subtypes, and one study (2.3%) did not specify a subtype. This distribution underscores the particular relevance of ferroptosis in TNBC, which aligns with the clinical challenge of chemoresistance in this aggressive subtype.

**Figure 2:**
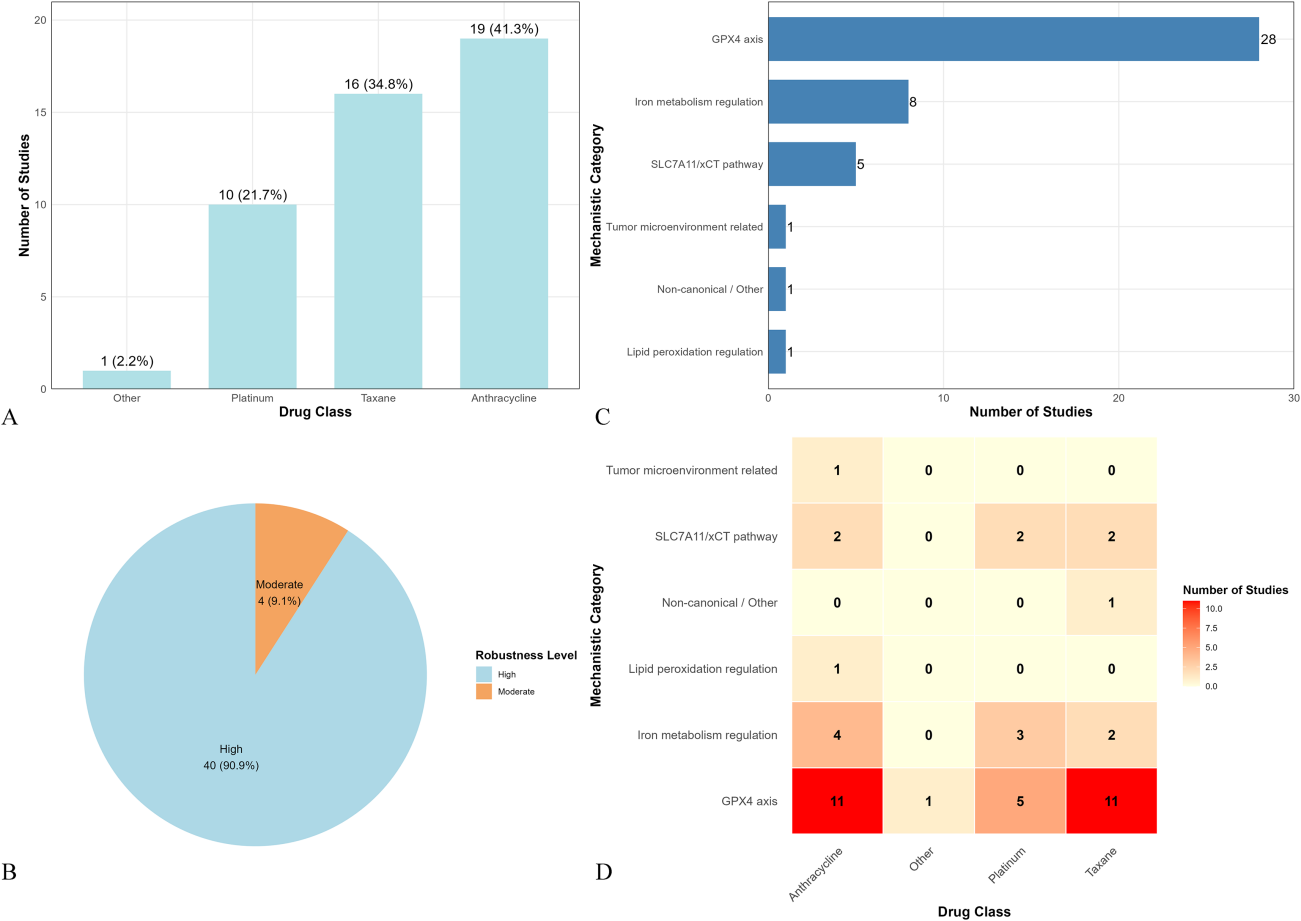
Overview of included studies and ferroptosis mechanisms. **(A)** Distribution of chemotherapeutic drug classes (anthracyclines, taxanes, platinum−based agents). Some studies investigated multiple drug classes. **(B)** Methodological quality assessment showing the proportion of studies with High, Moderate, or Limited robustness based on five quality indicators. **(C)** Distribution of the six mechanistic categories across the 44 included studies. The GPX4 axis predominates (31 studies, 70.5%). **(D)** Heatmap illustrating the number of studies investigating each ferroptosis mechanism within each drug class. The color intensity correlates with study count.

In terms of chemotherapeutic agents, anthracyclines (doxorubicin/adriamycin) were investigated in 19 studies (45.5%), taxanes (paclitaxel) in 16 studies (36.4%), and platinum−based drugs (cisplatin/carboplatin) in 10 studies (21.7%). Some studies investigated more than one drug class (**Figure 2A**). This distribution reflects the clinical landscape of breast cancer chemotherapy, with anthracyclines and taxanes representing the backbone of treatment regimens.

The experimental systems employed across studies were predominantly combined *in vitro* and *in vivo* approaches (35 studies, 79.5%), while nine studies (20.5%) were limited to *in vitro* investigations. Regarding resistance models, 41 studies (93.2%) established acquired resistance models through continuous drug exposure, and several studies analysed clinical resistant samples. The main characteristics of the 44 included studies are summarised in **Table 2**. Detailed characteristics and key mechanistic findings are provided in **Supplementary Tables 1**, **2**, respectively.

**Table 2:** Characteristics of included studies investigating ferroptosis−related chemoresistance in breast cancer.

Study	Year	Breast cancer subtype	Resistant model	Chemotherapeutic agent	Ferroptosis mechanism/target	*In vivo* validation	Main finding
Yu, X. et al. (31)	2024	HER2-negative	Acquired (clinical TAC-resistant samples)	Adriamycin	HIF1α/TFRC/Fe^2+^	Yes	HIF1α upregulates TFRC, increasing Fe^2+^ and inducing ferroptosis; overexpression reverses ADR resistance.
Sang, M. et al. (32)	2019	Mixed	EMT-induced (TGF-β1 stimulated)	Paclitaxel, Adriamycin	GPX4/xCT/GSH/Fe^2+^	Yes	Multifunctional nano-device induces ferroptosis via GSH depletion, Fenton reaction, and LPO burst, overcoming EMT-mediated resistance.
Zhang, X. et al. (33)	2025	Mixed	Acquired (Paclitaxel-resistant MCF-7PR/MDA-MB-231PR)	Paclitaxel	KHSRP/CISD1	Yes	A novel taxane overcomes paclitaxel resistance by binding to KHSRP, downregulating CISD1, and disrupting iron homeostasis to induce non-canonical ferroptosis.
Chen, Y. et al. (34)	2023	TNBC	Intrinsic (4T1)	Camptothecin	GPX4/Ferrocene/RSL3	Yes	A self-assembly nano-prodrug co-delivering CPT, ferrocene and RSL3 overcomes chemotherapy resistance by synergistically inducing ferroptosis and apoptosis in TNBC.
Liu, X. et al. (35)	2026	TNBC	Acquired (Doxorubicin-resistant 231R/1937R)	Doxorubicin	AMPKα2/NCOA4/FTH1	Yes	AMPKα2 upregulation promotes NCOA4 degradation, suppressing ferritinophagy, increasing FTH1, reducing labile iron pool, and inhibiting ferroptosis.
Yang, X. et al. (36)	2025	Mixed	Intrinsic	Paclitaxel	GPX4/CREB1/ASCL1	Yes	Inhibition of ASCL1 increases paclitaxel sensitivity by activating ferroptosis via the CREB1/GPX4 axis in breast cancer.
Chen, S. et al. (37)	2026	Mixed	Acquired (MCF-7/ADR)	Doxorubicin	Arachidonic acid/lipid peroxidation	Yes	Arachidonic acid-functionalized micelle induces lipid peroxidation and ferroptosis, synergizing with DOX to overcome resistance.
Peng, L. et al. (38)	2025	TNBC	Acquired (clinical relapsed samples)	Cisplatin	CLEC3B/SLC39A8/14	No	CLEC3B enhances cisplatin sensitivity by interacting with metal transporters to promote iron accumulation and ferroptosis.
Zhang, K. et al. (39)	2025	TNBC	Acquired (CAFs co-culture)	Doxorubicin	ZFP64/GCH1/FTH1	Yes	CAFs-derived lactate promotes ZFP64-mediated GCH1/FTH1 activation, suppressing ferroptosis and conferring DOX resistance.
Yu, X. et al. (40)	2024	Mixed	Acquired (MCF-7/ADR)	Adriamycin (Doxorubicin)	TFRC/Iron metabolism	No	TFRC upregulation increases Fe^2+^ accumulation and lipid peroxidation, activates ferroptosis via an iron-dependent pathway, and reverses adriamycin resistance in breast cancer cells.
He, Y. et al. (41)	2025	TNBC	Intrinsic (MDA-MB-231, HCC1937)	Cisplatin	DCTPP1/FOXA1/GPX4	Yes	FOXA1-activated DCTPP1 suppresses ferroptosis to promote TNBC progression and reduce cisplatin sensitivity.
Chatterjee, A. et al. (42)	2024	TNBC	Acquired (Cisplatin-resistant 4T1)	Cisplatin	TrxR1/GPX4	Yes	Sunshinamide dually targets TrxR1 and GPX4, inducing both apoptosis and ferroptosis to overcome cisplatin resistance.
Choi, H. et al. (43)	2025	TNBC	Acquired (HCC1806R, paclitaxel-resistant)	Doxorubicin/Cisplatin	xCT (SLC7A11)/GSH/Ferroptosis	Yes	Dual inhibition of glutaminase (GLS) and xCT depletes glutathione, disrupts redox balance, and overcomes chemoresistance in glutamine-dependent TNBC.
Shen, M. et al. (44)	2024	Mixed	Acquired (clinical non-pCR samples)	Doxorubicin	DNAJC12/AKT/GPX4/SLC7A11	Yes	DNAJC12 activates AKT to upregulate GPX4 and SLC7A11, suppressing DOX-induced ferroptosis and apoptosis.
Loftus, L.V. et al. (45)	2025	Mixed	Acquired (post-chemotherapy surviving cells)	Cisplatin/Docetaxel	GPX4/Labile iron/PCBP1	No	Chemotherapy-surviving cancer cells accumulate labile iron due to PCBP1 loss, creating a shared vulnerability to GPX4 inhibition-induced ferroptosis.
Perera, L. et al. (46)	2025	Mixed	Acquired (ABCB1/ABCG2-expressing)	Adriamycin	Erastin/RSL3/GPX4	Yes	Erastin and RSL3 overcome ABC transporter-mediated MDR by inhibiting GPX4 and enhancing chemotherapy uptake.
Zeng, W. et al. (47)	2025	Mixed	Acquired (clinical chemoresistant samples)	Doxorubicin, Docetaxel, Cyclophosphamide	MBOAT1/4-HNE	Yes	Ferroptotic neutrophils suppress CD8+ T cells via PGE2/IDO/oxidized lipids, driving immunosuppression and chemoresistance.
Zheng, Y. et al. (48)	2025	TNBC	Acquired (Paclitaxel-resistant 4T1/PTX)	Paclitaxel	GPX4/GSH/ROS/MDA	Yes	Mangiferin-based nanomedicine reverses paclitaxel resistance by downregulating GPX4, depleting GSH, and deactivating CAFs.
Zhu, Z. et al. (49)	2023	Mixed	Acquired (clinical non-pCR samples)	Doxorubicin	GATA3/CYB5R2/Fe^2+^	Yes	GATA3 represses CYB5R2, reducing Fe^2+^ availability, suppressing iron accumulation and ferroptosis, and driving DOX resistance.
Gong, S. et al. (50)	2026	Mixed	Acquired (MCF-7/ADM)	Doxorubicin	GSH/GPX4	Yes	GSH-responsive nanoinducer depletes GSH, downregulates GPX4, and reduces DOX IC50 by 147-fold in resistant cells.
Li, H. et al. (51)	2022	TNBC	Intrinsic (MDA-MB-231, HCC1937)	Cisplatin	HLF/GGT1/GPX4	Yes	TAM-derived TGF-β1 activates HLF via SMAD3, which transactivates GGT1 to suppress ferroptosis and drive TNBC progression and cisplatin resistance.
Liang, Y. et al. (52)	2023	Mixed	Acquired (MDA-MB-231/DOX)	Doxorubicin	HSPB1/ROS/MDA	Yes	HSPB1 activates NF-κB signaling to reduce ROS and MDA accumulation, inhibiting ferroptosis and conferring DOX resistance.
Chaudhary, N. et al. (53)	2025	TNBC	Acquired (drug-tolerant persister cells)	Paclitaxel, Doxorubicin, Cisplatin	GPX4/FSP1	Yes	DTP cells exhibit low GPX4 but adaptively upregulate FSP1; combined GPX4/FSP1 inhibition synergistically eradicates resistant cells.
Duan, W. et al. (54)	2024	Mixed	Intrinsic	Paclitaxel	Keap1/Nrf2/SLC7A11/GPX4	Yes	miR-141-3p targets Keap1 to activate Nrf2/SLC7A11/GPX4 axis, suppressing ferroptosis and promoting paclitaxel resistance in breast cancer.
Hao, T. et al. (55)	2025	Mixed	Acquired (MCF-7/DOX)	Doxorubicin	GPX4/GSH/ROS	Yes	Mitochondria-targeted microneedle system triggers ROS overproduction, GSH depletion, and GPX4 inactivation, inducing synergistic ferroptosis and apoptosis.
Huang, L. et al. (56)	2025	TNBC	Acquired (MDA-MB-231/ADR, MDA-MB-468/ADR)	Doxorubicin	ZC3H13/KCNQ1OT1/TRABD	Yes	ZC3H13-mediated m6A modification suppresses TRABD expression, increasing iron accumulation and reversing DOX resistance.
Zhang, C. et al. (57)	2025	Luminal	Acquired (MCF-7/ADR)	Doxorubicin	AKR1B1/STAT3/SLC7A11	Yes	Aldose reductase inhibitor 5a induces ferroptosis via AKR1B1/STAT3/SLC7A11 axis and inhibits ABCB1 to increase DOX accumulation.
Cao, G. et al. (58)	2026	TNBC	Acquired (MDA-MB-231/CDDP)	Cisplatin	xCT/GPX4	Yes	Platinum(IV) complex releases oxaliplatin and sulfasalazine derivative, inhibiting xCT/GPX4 axis, depleting GSH, and inducing ferroptosis.
Duan, W. et al. (59)	2025	Mixed	Intrinsic	Paclitaxel	SLC7A11/GPX4/AKT/mTOR	Yes	CAF-derived NRG1 activates AKT/mTOR to upregulate SLC7A11/GPX4, suppressing ferroptosis and driving paclitaxel resistance, while cancer cell-derived PDGFC forms a positive feedback loop activating CAFs.
Lin, C. et al. (60)	2025	TNBC	Acquired (MCF-7/Taxol)	Paclitaxel	GPX4/GSH/LPO	Yes	Peptide-based nanoassembly with laser irradiation generates ROS, depletes GSH, downregulates GPX4, and amplifies ferroptosis.
Yang, Y. et al. (61)	2022	Mixed	Acquired (MCF-7-Adr)	Paclitaxel, Adriamycin	IRP2/TfR1/Ferritin	Yes	Phenazine derivatives sequester iron in lysosomes, dysregulate iron homeostasis, and trigger ferroptosis to attenuate cancer stemness.
Maimon, A. et al. (62)	2025	TNBC	Acquired (analysis of chemoresistant patients)	Doxorubicin	PNKP/GPX4/FTH/SCD1	Yes	PNKP targeting induces ferroptosis via STING-mediated autophagy and STAT3 inhibition, synergizing with DOX.
Yuan, J. et al. (63)	2025	TNBC	Intrinsic (MDA-MB-231, MDA-MB-468)	Paclitaxel	GPX4/NF-κB	Yes	RSL3 activates NF-κB signaling to induce ferroptosis, enhancing TNBC chemosensitivity to paclitaxel *in vitro* and *in vivo*.
Li, M. et al. (64)	2025	Mixed	Intrinsic	Doxorubicin	GPX4	Yes	RSL3 induces ferroptosis-dependent ROS accumulation that disrupts glycolysis, thereby mitigating doxorubicin resistance in breast cancer cells.
Song, X. et al. (65)	2024	TNBC	Acquired (MDA-MB-468/DDP)	Cisplatin	SRSF1/circSEPT9/GCH1/SLC7A11	No	SRSF1 upregulates circSEPT9, which blocks GCH1 ubiquitination, suppressing ferroptosis and reducing cisplatin sensitivity.
Wang, J. et al. (66)	2025	TNBC	Intrinsic (MDA-MB-231, HCC1806, etc.)	Paclitaxel	GPX4/FTH1/CD24	Yes	STT3-mediated aberrant N-glycosylation of CD24 inhibits paclitaxel-induced ferroptosis by suppressing lipid peroxidation and iron accumulation, reducing chemosensitivity in TNBC.
Kuo, H. et al. (67)	2025	TNBC	Acquired (T50R, MDA-MB-231-derived)	Paclitaxel	SLC7A11/GPX4/Ferroptosis	No	SYK overexpression enhances microtubule instability, spindle abnormalities, and ER stress, leading to ferroptosis in paclitaxel-resistant TNBC cells when cultured without drug.
Ye, L. et al. (68)	2025	TNBC	Acquired (CSCs with P-gp overexpression)	Doxorubicin	GPX4/FSP1/ACSL4	Yes	Pravastatin depletes cholesterol, disrupts P-gp function, and synergizes with Fe_3_O_4_ to induce ferroptosis via GPX4/FSP1 downregulation.
Zhu, H. et al. (69)	2024	TNBC	Acquired (MDA-MB-231-R, MDA-MB-468-R)	Doxorubicin	MAGEA6/AMPKα1/SLC7A11	Yes	MAGEA6 silencing stabilizes AMPKα1, downregulates SLC7A11, and promotes ferroptosis, enhancing DOX sensitivity.
Wang, H. et al. (70)	2025	TNBC	Acquired (MDA-MB-231/Pac, SUM159PT/Pac)	Paclitaxel	miR-325-3p/GSTP1	Yes	miR-325-3p directly targets GSTP1; its overexpression restores paclitaxel sensitivity by inducing ferroptosis.
Tian, R. et al. (71)	2025	Mixed	Acquired (JIMT-1 trastuzumab-resistant)	Paclitaxel, Doxorubicin	PRSS3/PAR2/ERK1/2/SLC7A11/GPX4	Yes	PRSS3 knockdown induces ferroptosis through SLC7A11/GPX4 downregulation and TfR1 upregulation, sensitizing cells to chemotherapy.
Liu, X. et al. (72)	2024	TNBC	Intrinsic (MDA-MB-231, SUM159)	Paclitaxel	SLC7A11/OTUD5	Yes	OTUD5 deubiquitinates and stabilizes SLC7A11 to suppress ferroptosis, promoting TNBC progression and reducing paclitaxel sensitivity.
Li, H. et al. (73)	2025	TNBC	Acquired (MDA-MB-231/CDDP)	Cisplatin	UCHL1	No	UCHL1 knockdown induces ferroptosis (↑lipid ROS/MDA/Fe^2+^, ↓GSH), reduces IC50, and increases apoptosis in resistant cells.
Zeng, Y. et al. (74)	2025	TNBC	Intrinsic (MDA-MB-231, BT-549)	Carboplatin	FDFT1/GPX4/SLC7A11/ACSL4	Yes	THEM6 stabilizes FDFT1 by inhibiting K48-linked ubiquitination to promote ferroptosis, thereby enhancing carboplatin sensitivity in TNBC.

### 3.2 Risk of bias and methodological quality assessment

The methodological rigor of the 44 included preclinical studies was systematically evaluated using two complementary tools. For *in vivo* animal experiments, the SYRCLE Risk of Bias tool was applied. For *in vitro* experiments, a quality assessment tool adapted from the OHAT framework was used. All assessments were performed independently by two reviewers, with disagreements resolved through consensus. The detailed domain-by-domain scoring for each study is available in **Supplementary Table 3** (SYRCLE) and **Supplementary Table 4** (OHAT).

#### 3.2.1 *In vivo* quality assessment (SYRCLE tool)

Among the 44 included studies, 35 (79.5%) incorporated *in vivo* animal experiments and were assessed using the SYRCLE tool. Scores ranged from 4 to 6 out of a maximum of 8, with a median score of 6. Based on the predefined thresholds, all 35 studies (100%) were classified as having a moderate risk of bias. This uniform classification reflects common, well−documented limitations in preclinical animal research reporting, particularly the lack of detailed methodological descriptions, rather than fundamental flaws in experimental design.

Key methodological strengths included clear reporting of baseline characteristics (100%), appropriate control groups (100%), data integrity (100%), statistical methods (100%), and outcome consistency (100%). However, specific areas for improvement were identified. First, while 28 studies (80.0%) mentioned random group allocation, only 4 studies (11.4%) explicitly described the randomization method (e.g., random number table, computer−generated sequence). Second, allocation concealment and blinding were the least reported items. No study (0%) clearly described allocation concealment, and blinding of investigators during outcome assessment was also not reported in any study (0%). The absence of blinding represents a potential source of performance and detection bias.

#### 3.2.2 *In vitro* quality assessment (OHAT-adapted framework)

The quality of the *in vitro* experiments from all 44 studies was uniformly high. Using the adapted OHAT criteria (scores ranging from 0-7), all 44 studies (100%) achieved a low risk of bias classification, with scores ranging from 6 to 7 (median: 7). Among these, 42 studies (95.5%) achieved a perfect score of 7/7, and the remaining 2 studies (4.5%) scored 6/7 (see **Supplementary Table 4**). This high level of methodological rigor was consistently demonstrated across several key domains. All studies (100%) clearly reported drug concentrations, treatment durations, and solvents used. Every study (100%) utilised multiple, complementary assays (e.g., CCK−8 for viability combined with flow cytometry for apoptosis and lipid ROS detection) to validate their conclusions. All studies reported appropriate statistical tests and provided precise p−values or confidence intervals, and all studies stated that key experiments were performed with at least three independent replicates. The two studies with a score of 6/7 (31, 32) lacked clear statements on authenticated cell lines or routine mycoplasma testing, although all other methodological criteria were met.

#### 3.2.3 Summary of methodological robustness

Rescue experiments using specific ferroptosis inhibitors (ferrostatin-1, liproxstatin-1) or iron chelators (deferoxamine) were performed in 39 studies (88.6%), providing supportive evidence for ferroptosis involvement in chemosensitivity modulation. *In vivo* validation was reported in 35 studies (79.5%), with xenograft or orthotopic mouse models confirming the translational relevance of mechanistic findings. Acquired resistance models were successfully established in 41 studies (93.2%), predominantly through continuous exposure to increasing drug concentrations over 3–6 months. Further analysis of specific ferroptosis-related experimental features revealed that GPX4 modulation was investigated in 31 studies (70.5%), SLC7A11 modulation in 14 studies (31.8%), and both pathways in 10 studies (22.7%) (**Supplementary Figure 2**).

The comprehensive methodological robustness assessment, based on five quality indicators (lipid peroxidation measurement, iron assessment, rescue experiment, *in vivo* validation, and statistical methods), classified 40 studies (90.9%) as having high robustness (**Figure 2C**). While *in vivo* studies could be strengthened by better adherence to reporting standards for randomization, blinding, and allocation concealment, the *in vitro* data provide a highly reliable foundation for the mechanistic conclusions. The high proportion of studies meeting key quality indicators supports the reliability of the synthesized findings regarding ferroptosis-mediated reversal of chemoresistance.

### 3.3 Mechanistic classification of ferroptosis regulation

The 44 included studies were categorized into six mechanistic classes based on their primary ferroptosis regulatory pathways (**Table 3**, **Figure 2A**). The distribution was as follows:

**Table 3:** Mechanistic classification of ferroptosis regulatory pathways.

Mechanistic_category	Count	Percentage (%)
GPX4 axis	28	63.6
Iron metabolism regulation	8	18.2
SLC7A11/xCT pathway	5	11.4
Lipid peroxidation regulation	1	2.3
Tumor microenvironment related	1	2.3
Non-canonical/Other	1	2.3
Total	44	100

#### 3.3.1 GPX4 axis (28 studies, 63.6%)

This was the predominant mechanism, involving direct or indirect modulation of glutathione peroxidase 4, the key enzyme that prevents lipid peroxidation. Studies demonstrated that downregulation of GPX4 through genetic or pharmacological interventions consistently sensitized resistant cells to chemotherapy.

#### 3.3.2 Iron metabolism regulation (8 studies, 18.2%)

These investigations focused on proteins controlling iron uptake (TFR1), storage (FTH1), export (FPN), or ferritinophagy (NCOA4). Modulation of these targets altered the labile iron pool, thereby influencing Fenton reaction-mediated lipid peroxidation.

#### 3.3.3 SLC7A11/xCT pathway (5 studies, 11.4%)

This mechanism involves the cystine/glutamate antiporter, which provides cysteine for glutathione synthesis. Inhibition of SLC7A11 depleted glutathione, impaired GPX4 function, and promoted ferroptosis.

#### 3.3.4 Other mechanisms (3 studies, 6.8%)

The remaining three studies explored lipid peroxidation regulation (arachidonic acid supplementation), tumor microenvironment−related mechanisms (CAF−derived lactate promoting histone lactylation), and a non−canonical pathway involving KHSRP/CISD1−mediated iron homeostasis disruption.

**Figure 3:**
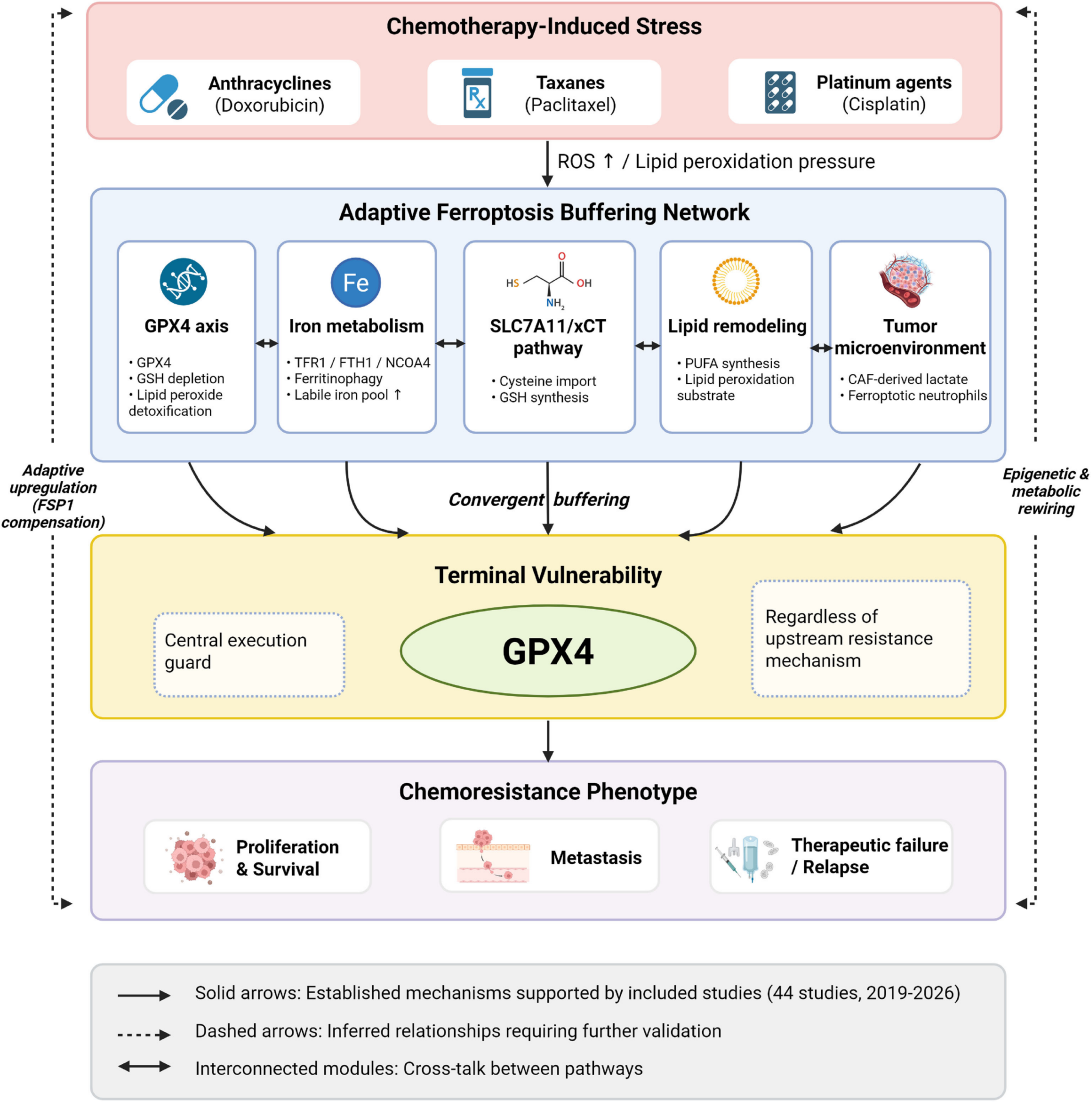
Adaptive ferroptosis buffering network in chemotherapy−resistant breast cancer (Created in https://BioRender.com).

The predominance of GPX4 axis and iron metabolism regulation indicates that these two pathways represent the core ferroptosis regulatory networks exploited to overcome chemoresistance in breast cancer. To illustrate how these diverse mechanisms integrate into a unified resistance framework, we propose a conceptual model of the adaptive ferroptosis buffering network (**Figure 3**).

### 3.4 Cross-analysis of mechanisms and drug classes

The association between ferroptosis mechanisms and chemotherapeutic drug classes revealed distinct patterns (**Table 4**, **Figure 2D**). The GPX4 axis was investigated across all drug classes, with 11 studies in anthracyclines, 11 in taxanes, and 5 in platinum−based drugs, demonstrating its broad relevance. Iron metabolism regulation was also distributed across multiple drug classes (4 anthracycline, 3 platinum, 2 taxane). The SLC7A11/xCT pathway showed involvement in 2 anthracycline, 2 platinum, and 2 taxane studies. Notably, anthracyclines were associated with the most diverse mechanistic landscape, involving all six categories, while taxanes and platinum-based drugs showed more focused mechanistic profiles. This may reflect the distinct mechanisms of action of these drug classes and the corresponding resistance pathways that cancer cells develop.

**Table 4:** Cross-analysis of ferroptosis mechanisms and chemotherapeutic drug classes.

Mechanism	Anthracycline	Platinum	Taxane	Other	Total
GPX4 axis	11	5	11	1	28
Iron metabolism regulation	4	3	2	0	9
SLC7A11/xCT pathway	2	2	2	0	6
Lipid peroxidation regulation	1	0	0	0	1
Non-canonical/Other	0	0	1	0	1
Tumor microenvironment related	1	0	0	0	1

Some studies investigated more than one drug class and therefore appear in multiple columns. The “Other” drug class includes camptothecin.

### 3.5 Evidence strength ranking of ferroptosis mechanisms

To integrate quantitative, methodological, and translational dimensions, we developed an evidence strength ranking system based on three criteria: number of studies, proportion with *in vivo* validation, and breadth of drug classes covered. The ranking algorithm classified mechanisms into three evidence levels (**Table 1**).

The GPX4 axis emerged as the strongest evidence mechanism, with 28 studies, 96.4% *in vivo* validation, and coverage across four drug classes (anthracyclines, taxanes, platinum, and other), earning a Level A classification. Iron metabolism regulation also achieved Level A, with 8 studies, 50.0% *in vivo* validation, and broad drug class coverage (all three major classes). Notably, the SLC7A11/xCT pathway was also classified as Level A in the updated analysis, with 5 studies and 80.0% *in vivo* validation across three drug classes.

The remaining mechanisms—lipid peroxidation regulation, non−canonical pathway, and tumor microenvironment−related—were each represented by single studies with single drug class coverage, receiving Level C (preliminary evidence) classifications.

This evidence hierarchy provides a framework for prioritizing mechanisms for clinical translation, with the GPX4 axis, iron metabolism regulation, and the SLC7A11/xCT pathway representing the most robustly supported targets for overcoming chemotherapy resistance in breast cancer.

### 3.6 Efficacy of ferroptosis-mediated chemosensitization

The 44 included studies were categorized into three major mechanistic groups based on their primary ferroptosis regulatory pathway: GPX4 axis (28 studies), iron metabolism regulation (8 studies), and SLC7A11/xCT pathway (5 studies). Three additional studies explored lipid peroxidation regulation, tumour microenvironment−related mechanisms, and non−canonical pathways (**Table 5**).

**Table 5:** Summary of ferroptosis−targeting studies by mechanism.

Mechanism	Intervention strategy	n	Key outcomes	Rescue (%)	*In vivo* (%)
GPX4 axis	GPX4 knockdown/knockout	12	IC50 ↓, ROS ↑, LPO ↑	83%	75%
	GPX4 inhibitors (RSL3, FIN56)	15	IC50 ↓, GPX4 ↓, lipid ROS ↑	100%	87%
	Nanoformulations targeting GPX4	8	IC50 ↓, tumor growth ↓, GSH ↓	88%	100%
	Combined GPX4/FSP1 inhibition	2	Synergistic ferroptosis	100%	100%
Iron metabolism	TFR1/HIF1α modulation	5	Fe^2+^ ↑, LPO ↑, IC50 ↓	80%	80%
	Ferritinophagy (NCOA4/FTH1)	4	Labile iron pool ↑, ferritin ↓	100%	75%
	Iron transporters (SLC39A8/14)	3	Fe^2+^ ↑, cisplatin sensitivity ↑	100%	67%
	Lysosomal iron sequestration	2	Fe^2+^ ↑, lipid ROS ↑, stemness ↓	100%	100%
	Other (UCHL1, PCBP1, GATA3)	2	Fe^2+^ ↑, GPX4 independent	100%	50%
SLC7A11/xCT	SLC7A11 knockdown/inhibitors	6	GSH ↓, lipid ROS ↑, drug sensitivity ↑	100%	83%
	Epigenetic/transcriptional regulation	3	SLC7A11 ↓, ferroptosis ↑	100%	67%
	Combined xCT + GLS inhibition	1	Glutathione depletion	100%	100%
Other	Lipid peroxidation induction	1	LPO ↑, synergizes with DOX (37)	Yes	Yes
	Tumour microenvironment (ferroptotic neutrophils)	1	Immunosuppression, chemoresistance (47)	N/A	Yes
	Non-canonical (KHSRP/CISD1)	1	Iron homeostasis disruption (33)	No	Yes

Some studies investigated multiple mechanisms and may appear in more than one row. Rescue experiment: use of ferrostatin−1, liproxstatin−1, or deferoxamine. *In vivo*: animal experiment performed.

#### 3.6.1 Representative findings

Across all mechanisms, ferroptosis induction consistently reduced chemoresistance *in vitro* and *in vivo*, as evidenced by decreased IC_50_ values, increased lipid peroxidation, and reversal of effects by ferroptosis inhibitors (ferrostatin−1, liproxstatin−1) or iron chelators (deferoxamine). Combined targeting of GPX4 with FSP1 (53), or integration of chemotherapy with ferroptosis−inducing nanoformulations (32, 60), showed synergistic efficacy in eradicating drug−tolerant persister cells and cancer stem−like cells. Mechanistically, iron metabolism regulation via the HIF1α−TFRC axis (31, 40) and SLC7A11 modulation via multiple upstream regulators (54, 59, 65, 72) represent convergent adaptive responses that can be therapeutically exploited. While most strategies target the GPX4−GSH axis, emerging non−canonical pathways (33) and tumour microenvironment−mediated mechanisms (47) warrant further investigation.

### 3.7 Synthesis of evidence and translational implications

This systematic review synthesized evidence from 44 preclinical studies investigating ferroptosis−mediated chemosensitization in breast cancer. The GPX4 axis was identified in 28 studies (63.6%), reflecting its central role as a terminal vulnerability, followed by iron metabolism regulation in 8 studies (18.2%) and the SLC7A11/xCT pathway in 5 studies (11.4%). Three additional mechanisms—lipid peroxidation regulation, tumor microenvironment−related pathways, and non−canonical mechanisms—were each represented by a single study (2.3% each).

Cross−analysis revealed that GPX4 modulation demonstrated efficacy across all three major drug classes (anthracyclines, taxanes, and platinum−based agents), while iron metabolism regulation and the SLC7A11/xCT pathway also showed broad applicability across multiple drug classes. An evidence strength ranking system (**Table 1**) classified the GPX4 axis, iron metabolism regulation, and the SLC7A11/xCT pathway as Level A (strong evidence), and the remaining mechanisms as Level C (preliminary evidence).

Methodologically, the included studies demonstrated adequate rigor for preclinical systematic reviews. Ferroptosis rescue experiments were performed in 39 studies (88.6%), and *in vivo* validation was reported in 35 studies (79.5%), supporting the causal involvement of ferroptosis in chemosensitization. Detailed methodological characteristics are summarised in **Supplementary Tables 1**–**4**.

## 4 Discussion

### 4.1 Summary of key findings

This systematic review of 44 preclinical studies provides the first comprehensive synthesis of evidence demonstrating that ferroptosis regulation is a convergent and therapeutically actionable mechanism to overcome chemotherapy resistance in breast cancer. Through a structured narrative synthesis and an evidence strength ranking system, we identified the GPX4 axis, iron metabolism regulation, and the SLC7A11/xCT pathway as the most robustly supported mechanisms (all Level A), followed by three additional mechanisms with preliminary evidence (Level C). The methodological quality of the included studies was adequate for preclinical systematic reviews, with rescue experiments performed in 39 studies (88.6%) and *in vivo* validation reported in 35 studies (79.5%). These findings provide supportive evidence that ferroptosis is a contributory mechanism underlying chemosensitization, although the preliminary nature of the evidence base should be acknowledged.

### 4.2 Mechanistic insights and biological plausibility

**Figure 3** provides a visual synthesis of this adaptive network. As depicted, chemotherapy−induced oxidative stress activates five interconnected buffering modules that converge on GPX4, which acts as a terminal vulnerability node regardless of the upstream resistance mechanism. The predominance of the GPX4 axis (58.6%) as a resistance mechanism is biologically grounded: GPX4 serves as the master regulator of ferroptosis, catalyzing the reduction of lipid hydroperoxides to non-toxic lipid alcohols and thereby preventing lethal lipid peroxidation (75). In chemoresistant cancer cells, upregulation of GPX4 or its upstream regulators represents an adaptive survival mechanism against oxidative stress induced by chemotherapeutic agents (76). Regardless of the primary resistance mechanism, cancer cells ultimately rely on GPX4 to mitigate oxidative damage. The consistent finding that GPX4 inhibition restores chemosensitivity across anthracycline, taxane, and platinum resistance supports the concept of GPX4 as a “terminal vulnerability”—a universal and compelling therapeutic target.

Iron metabolism regulation (16 studies, 36.4%) operates upstream of lipid peroxidation. By modulating iron uptake (TFR1), storage (FTH1), and ferritinophagy (NCOA4), these strategies increase the labile iron pool available for Fenton chemistry, amplifying lipid peroxidation and ferroptosis (77). The identification of AMPKα2-mediated NCOA4 phosphorylation (68) and the development of iron-based nanoplatforms (71) exemplify innovative approaches that integrate iron homeostasis with other cellular processes such as autophagy and cholesterol metabolism. The convergence of these pathways highlights the complexity of ferroptosis regulation and underscores the rationale for multi−target strategies, though such approaches require careful safety evaluation.

The SLC7A11/xCT pathway (10 studies, 22.7%) provides a direct link between amino acid metabolism and ferroptosis. By controlling cysteine import for glutathione synthesis, SLC7A11 acts as a gatekeeper of cellular antioxidant capacity (78). Its inhibition, whether through miR-325-3p-mediated GSTP1 downregulation (70) or novel platinum(IV) complexes (58), effectively depletes GSH, inactivates GPX4, and promotes ferroptosis. The availability of clinically approved xCT inhibitors (e.g., sulfasalazine) positions this pathway as potentially translatable (79), but preclinical optimization and toxicity studies remain necessary.

Mechanisms with preliminary evidence include lipid peroxidation regulation, tumor microenvironment-related pathways, and non-canonical mechanisms, all of which represent emerging frontiers. The finding that cancer-associated fibroblasts can promote chemoresistance through lactate-mediated epigenetic modulation (39) highlights the importance of considering the tumor microenvironment in ferroptosis-based therapeutic strategies. Similarly, the KHSRP/CISD1-mediated iron homeostasis disruption (33) opens new avenues for targeting mitochondrial iron handling, a relatively unexplored area in ferroptosis research. These single−study mechanisms require independent replication before their therapeutic potential can be reliably assessed.

### 4.3 Comparison with prior research

Our findings align with and extend previous systematic reviews on ferroptosis in cancer. While prior reviews have broadly discussed the role of ferroptosis in overcoming drug resistance across multiple cancer types, this is the first systematic review to focus specifically on breast cancer and to provide a mechanistic classification and evidence strength hierarchy. The predominance of TNBC-focused studies (47.7%) in our included literature corroborates the well-established vulnerability of TNBC to ferroptosis, attributed to its iron- and lipid-rich phenotype (61).

Our evidence ranking system extends previous qualitative discussions by providing a quantitative framework for prioritizing mechanisms for clinical translation. The Level A classification of the GPX4 axis, iron metabolism regulation, and the SLC7A11/xCT pathway is consistent with the broader ferroptosis literature, where these pathways are recognized as central regulators. Notably, the SLC7A11/xCT pathway, despite being supported by fewer studies (n=5, 11.4% of the evidence base), achieved Level A status due to its high proportion of *in vivo* validation (80%) and coverage across all three major drug classes, underscoring its translational potential. All of our findings are based exclusively on preclinical models, and the translational gap to clinical applications remains substantial.

### 4.4 Clinical implications and translational potential

The robust preclinical evidence summarized in this review has several important clinical implications, although considerable caution is warranted given the preliminary nature of the evidence base. First, the moderate−to−high proportion of studies with *in vivo* validation (79.5%) and rescue experiments (88.6%) provides supportive evidence for developing ferroptosis−inducing agents as adjuncts to conventional chemotherapy. However, most studies used cell line−derived xenografts, and validation in patient−derived models remains limited.

Second, several strategies show preliminary translational promise. Nanoformulations such as PTX@CPG (60) and Fe/CDP (68) demonstrate enhanced tumor targeting and reduced systemic toxicity in preclinical models. Repurposed drugs such as pravastatin, already FDA-approved for cardiovascular indications, offer a rapid pathway to clinical investigation. Natural compounds like phenazine derivatives (61) provide novel chemical scaffolds for ferroptosis induction. Nevertheless, none of these strategies have yet entered clinical trials, and significant safety and efficacy barriers remain to be addressed.

Third, the evidence hierarchy developed herein provides an exploratory roadmap for prioritizing mechanisms for clinical development. Level A mechanisms (the GPX4 axis and iron metabolism regulation) may warrant priority for first−in−class ferroptosis−inducing agents, given their relatively robust validation across multiple drug classes. Level B mechanisms, such as the SLC7A11/xCT pathway, merit further investigation, particularly given the availability of clinically approved inhibitors (e.g., sulfasalazine). Level C mechanisms, while scientifically intriguing, require independent replication and foundational research to define their therapeutic window before clinical consideration. Collectively, the path from preclinical promise to clinical application will require rigorous safety evaluation, predictive biomarker development, and well−designed early−phase trials.

### 4.5 Limitations of the evidence

Despite the supportive evidence base, several limitations must be acknowledged. First, most studies relied on cell line−derived xenografts (CDX), with only 35 of 44 studies (79.5%) including *in vivo* validation, and even fewer using patient−derived xenograft (PDX) models. CDX models, while valuable, may not fully recapitulate the heterogeneity and microenvironment of human tumors.

Second, potential publication bias cannot be excluded, as all included studies reported positive findings. Moreover, the rapid growth of publications in 2024−2026 (the majority of included studies) raises concern for “hot−topic” bias, where journals may preferentially publish positive results on emerging themes such as ferroptosis.

Third, the heterogeneity of breast cancer subtypes beyond TNBC remains underexplored. Only four studies (9.1%) focused on luminal or HER2−negative subtypes. This gap likely reflects the well−established intrinsic susceptibility of TNBC to ferroptosis due to its iron− and lipid−rich phenotype. However, it also suggests that ferroptosis regulation in hormone−dependent or HER2−driven breast cancers remains largely unexplored. Given the distinct metabolic and signaling landscapes of these subtypes, future studies should systematically evaluate whether lower ferroptosis susceptibility in luminal tumors is attributable to higher GPX4 expression, different lipid profiles, or alternative antioxidant mechanisms.

Fourth, the long−term safety and potential off−target effects of ferroptosis−inducing agents require systematic evaluation. GPX4 inhibitors, while effective, may induce ferroptosis in normal tissues (80). The development of tumor−targeted delivery systems, as exemplified by the nanoformulations in this review, represents a promising strategy to mitigate these risks, but such approaches remain at an early preclinical stage.

Fifth, the absence of standardized outcome measures across studies precluded quantitative meta−analysis. The substantial heterogeneity in study design, resistance models, ferroptosis assays, and reported outcomes (IC_50_, cell viability, tumor volume, etc.) made statistical pooling inappropriate. The development of consensus guidelines for ferroptosis research, including standardized assays for lipid peroxidation, iron measurement, and ferroptosis validation, would greatly enhance the comparability and reproducibility of future studies.

Finally, our risk of bias assessment revealed significant reporting deficiencies in *in vivo* studies, particularly in randomization (only 4 of 35 *in vivo* studies, 11.4%, explicitly described the method), allocation concealment (0%), and blinding (0%). While these limitations are common in preclinical research, they underscore the need for adherence to reporting guidelines such as ARRIVE to enhance the validity and reproducibility of findings.

### 4.6 Future directions

Based on the gaps identified in this review, we propose several priorities for future research. An urgent need exists for the development of specific and safe pharmacological tools, as many current studies rely on genetic manipulation. Highly specific, potent, and bioavailable small molecule inhibitors or inducers of key ferroptosis regulators—such as GPX4, FSP1, and NCOA4—with favorable safety profiles for *in vivo* use are essential. Structure-based drug design and high-throughput screening campaigns should be prioritized to accelerate the discovery of such compounds.

Another priority is the systematic exploration of combination strategies. Given that preclinical evidence strongly supports the combination of ferroptosis inducers with conventional chemotherapy, future studies should systematically evaluate optimal dosing schedules, sequence of administration, and potential synergistic combinations with other targeted therapies, including PARP inhibitors and CDK4/6 inhibitors, as well as immunotherapies such as immune checkpoint blockers. The interplay between ferroptosis and immunogenic cell death presents a particularly exciting avenue for investigation.

A third direction is the identification of predictive biomarkers to enable patient stratification in future clinical trials. Research must focus on identifying robust and easily measurable biomarkers of ferroptosis sensitivity, including the expression levels of GPX4, SLC7A11, ACSL4, and FTH1 in tumor biopsies; the labile iron pool in circulating tumor cells; and lipid peroxidation markers in plasma. Multi-omics approaches integrating genomics, transcriptomics, and metabolomics may reveal novel biomarker signatures that could guide clinical decision-making.

The expansion of research to non−TNBC subtypes represents another important priority. The underrepresentation of luminal and HER2−positive breast cancers in the current literature (only 9.1% of included studies) constitutes a critical gap, and future studies should systematically evaluate ferroptosis regulation in these subtypes, taking into account their distinct metabolic dependencies and resistance mechanisms.

Furthermore, validation in advanced preclinical models is essential to enhance translational relevance. Beyond conventional cell line-derived xenografts, future research should prioritize validation in patient-derived xenograft models, orthotopic models, and genetically engineered mouse models that more faithfully recapitulate tumor heterogeneity, microenvironment, and metastatic progression. The incorporation of co-clinical trials, where preclinical findings are validated in parallel with early-phase clinical investigations, could substantially accelerate the translation of ferroptosis-targeting strategies.

Finally, the integration of tumour microenvironment considerations into ferroptosis research is increasingly recognised as important. The preliminary evidence linking cancer-associated fibroblasts to ferroptosis resistance (81) highlights the need for deeper investigation into how stromal cells, immune cells, and the extracellular matrix modulate ferroptosis susceptibility. Advanced co-culture systems and single-cell technologies will be essential for dissecting these complex interactions and may reveal novel therapeutic opportunities.

## 5 Conclusion

In conclusion, this systematic review suggests that ferroptosis regulation represents a mechanistically diverse and preliminarily promising strategy to overcome chemotherapy resistance in breast cancer. Through our evidence hierarchy, we prioritize the GPX4 axis, iron metabolism regulation, and the SLC7A11/xCT pathway as the most consistently supported targets, providing a preliminary framework to guide future research and clinical translation. Nevertheless, given that all included evidence is derived from preclinical models, the translational gap to clinical applications remains substantial. Addressing key gaps, including the need for PDX validation, subtype−specific investigation, safety evaluation, and predictive biomarker development, will be essential to determine whether ferroptosis induction can be successfully translated into clinical practice. As the field continues to expand rapidly, we hope this foundational synthesis will help guide and accelerate progress toward meaningful clinical applications, while recognising that rigorous validation in human studies is still awaited.

## Data Availability

The original contributions presented in the study are included in the article/**Supplementary Material**. Further inquiries can be directed to the corresponding author.

## Supplementary material

Supplementary Figure 1 Annual distribution of included publications.

Supplementary Figure 2 Frequency of GPX4 and SLC7A11 modulation across included studies.

Supplementary Table 1 Characteristics of included studies.

Supplementary Table 2 Key mechanistic findings of included studies.

Supplementary Table 3 SYRCLE risk of bias assessment for *in vivo* studies.

Supplementary Table 4 Quality assessment for *in vitro* studies (adapted from OHAT framework).

Supplementary Table 5 Summary of methodological characteristics across included studies.

Supplementary File 1 Complete search strategies for PubMed, Scopus, Embase, and Web of Science Core Collection.

Supplementary File 2 Completed PRISMA 2020 checklist for this systematic review.
