# Supplementary figures and images for: Ferroptosis in chemotherapy resistance and resensitization in breast cancer: a systematic review of preclinical evidence and translational implications
Source: Front Oncol. 2026 Jul 1;16:1854602. doi: 10.3389/fonc.2026.1854602 (PMC13368658; doi:10.3389/fonc.2026.1854602)

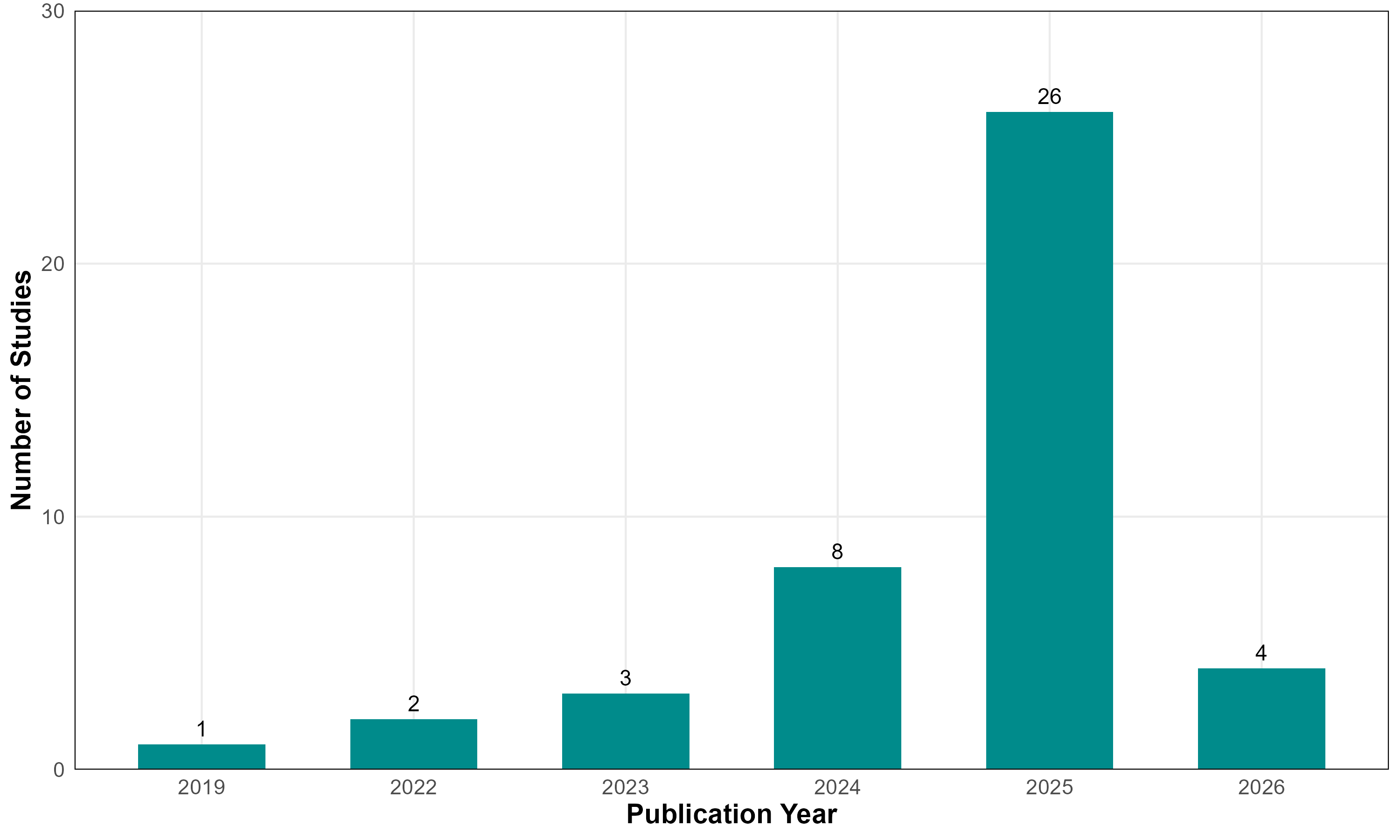

Supplement: Supplementary Figure 1 — Annual distribution of included publications. [file Image1.tif]

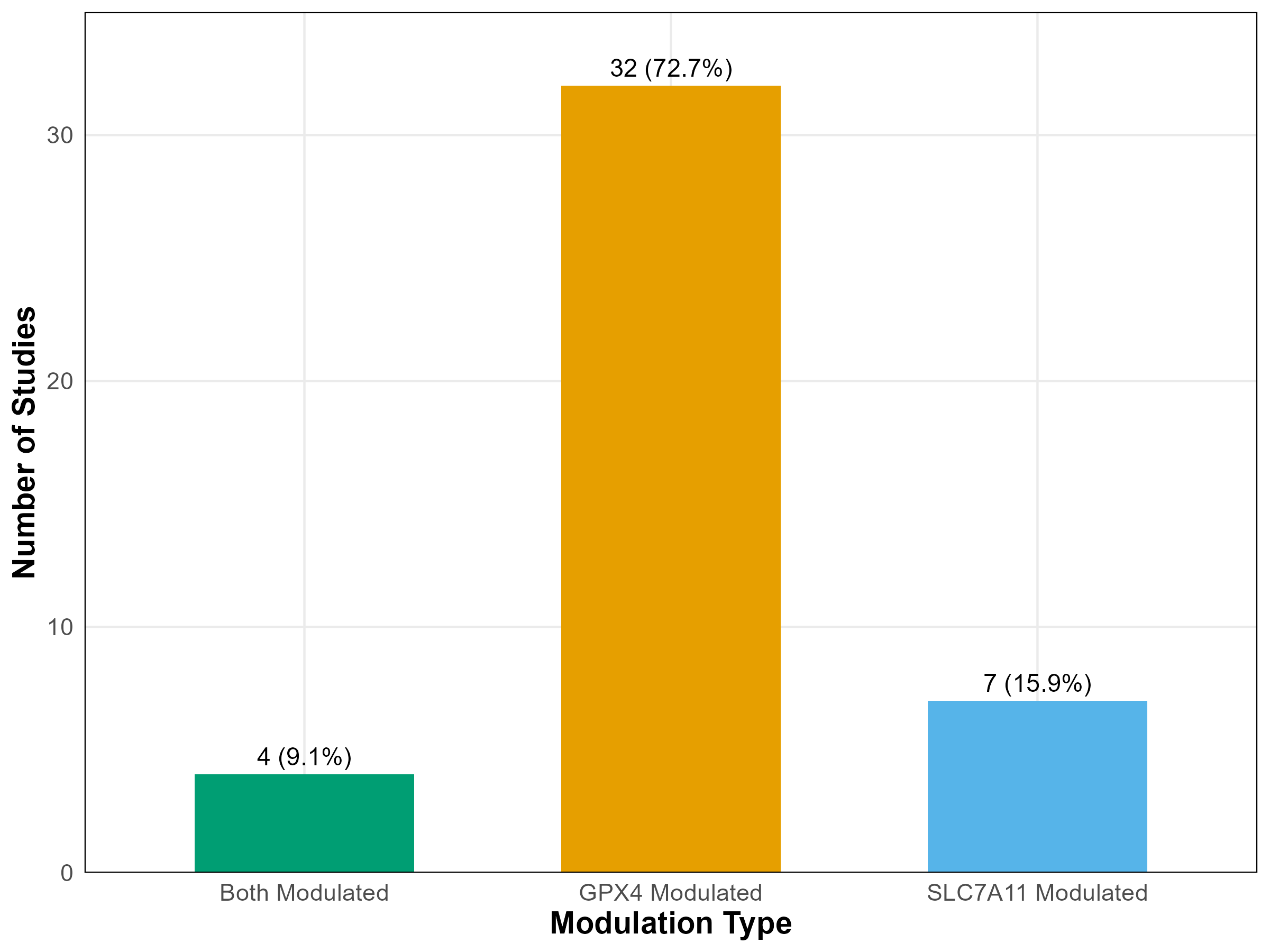

Supplement: Supplementary Figure 2 — Frequency of GPX4 and SLC7A11 modulation across included studies. [file Image2.tif]
