# Supplementary material for: Ferroptosis in chemotherapy resistance and resensitization in breast cancer: a systematic review of preclinical evidence and translational implications
Source: Front Oncol. 2026 Jul 1;16:1854602. doi: 10.3389/fonc.2026.1854602 (PMC13368658; doi:10.3389/fonc.2026.1854602)
Supplement: Supplementary File 1 — Complete search strategies for PubMed, Scopus, Embase, and Web of Science Core Collection. [file DataSheet1.docx]

**Complete search strings for PubMed, Scopus, Embase and Web of Science (May 2026)**

**Pubmed**

("Breast Neoplasms"[Mesh] OR "breast cancer"[tiab] OR "breast carcinoma"[tiab] OR "breast neoplasm*"[tiab])

AND

("Ferroptosis"[Mesh] OR ferroptosis[tiab] OR ferroptotic[tiab])

AND

("Drug Resistance, Neoplasm"[Mesh] OR chemoresistance[tiab] OR "chemotherapy resistance"[tiab] OR "drug resistance"[tiab] OR "therapy resistance"[tiab])

**Scopus**

( "breast cancer" OR "breast carcinoma" )

AND

( ferroptosis )

AND

( "chemotherapy resistance" OR chemoresistance OR "drug resistance" OR "therapy resistance" )

AND

( chemotherapy OR doxorubicin OR paclitaxel OR cisplatin OR anthracycline* OR taxane* )

**Embase**

('breast cancer'/exp OR 'breast cancer':ti,ab OR 'breast carcinoma':ti,ab)

AND

('ferroptosis'/exp OR ferroptosis:ti,ab OR ferroptotic:ti,ab)

AND

('drug resistance'/exp OR chemoresistance:ti,ab OR 'chemotherapy resistance':ti,ab OR 'drug resistance':ti,ab OR 'therapy resistance':ti,ab)

**Web of Science Core Collection**

TS=("breast cancer" OR "breast carcinoma")

AND

TS=(ferroptosis OR ferroptotic)

AND

TS=(chemoresistance OR "chemotherapy resistance" OR "drug resistance" OR "therapy resistance")
