# Supplementary material for: Ferroptosis in chemotherapy resistance and resensitization in breast cancer: a systematic review of preclinical evidence and translational implications
Source: Front Oncol. 2026 Jul 1;16:1854602. doi: 10.3389/fonc.2026.1854602 (PMC13368658; doi:10.3389/fonc.2026.1854602)
Supplement: Supplementary File 2 — Completed PRISMA 2020 checklist for this systematic review. [file DataSheet2.docx]

| **Section and Topic** | **Item #** | **Checklist item** | **Location where item is reported** |
| --- | --- | --- | --- |
| **TITLE** | | |  |
| Title | 1 | Identify the report as a systematic review. | Title page: "A Systematic Review of Preclinical Evidence and Translational Implications" |
| **ABSTRACT** | | |  |
| Abstract | 2 | See the PRISMA 2020 for Abstracts checklist. | Abstract (page 1‑2): Structured summary with Background, Methods, Results, Conclusion |
| **INTRODUCTION** | | |  |
| Rationale | 3 | Describe the rationale for the review in the context of existing knowledge. | Introduction (Section 1): Summarizes breast cancer burden, TNBC chemoresistance, ferroptosis biology, and lack of systematic synthesis |
| Objectives | 4 | Provide an explicit statement of the objective(s) or question(s) the review addresses. | Introduction (Section 1, paragraph 5): "What are the predominant ferroptosis regulatory mechanisms...? How do these mechanisms distribute...? Which mechanisms are supported by the most robust evidence...?" |
| **METHODS** | | |  |
| Eligibility criteria | 5 | Specify the inclusion and exclusion criteria for the review and how studies were grouped for the syntheses. | Section 2.2 "Inclusion and Exclusion Criteria" (pages 5); Section 2.6 "Data Synthesis": describes grouping by chemotherapeutic class and mechanistic categories |
| Information sources | 6 | Specify all databases, registers, websites, organisations, reference lists and other sources searched or consulted to identify studies. Specify the date when each source was last searched or consulted. | Section 2.1 "Search Strategy": PubMed, Scopus, Embase, Web of Science Core Collection; search date May 26, 2026; citation tracking of reference lists |
| Search strategy | 7 | Present the full search strategies for all databases, registers and websites, including any filters and limits used. | Section 2.1 "Search Strategy": full search terms and Boolean operators described; complete strategies provided in Supplementary File 1 |
| Selection process | 8 | Specify the methods used to decide whether a study met the inclusion criteria of the review, including how many reviewers screened each record and each report retrieved, whether they worked independently, and if applicable, details of automation tools used in the process. | Section 2.3 "Study Selection Process" (page 6): "Two reviewers independently conducted the screening process. Disagreements were resolved through discussion until consensus was reached." |
| Data collection process | 9 | Specify the methods used to collect data from reports, including how many reviewers collected data from each report, whether they worked independently, any processes for obtaining or confirming data from study investigators, and if applicable, details of automation tools used in the process. | Section 2.4 "Data Extraction": one reviewer extracted data using a standardized template, independently verified by a second reviewer; authors not contacted for missing data |
| Data items | 10a | List and define all outcomes for which data were sought. Specify whether all results that were compatible with each outcome domain in each study were sought (e.g. for all measures, time points, analyses), and if not, the methods used to decide which results to collect. | Section 2.4 "Data Extraction": outcomes extracted include IC50, cell viability, apoptosis assays, clonogenic survival, tumor growth inhibition, and ferroptosis markers (lipid peroxidation, iron, GSH, GPX4) |
|  | 10b | List and define all other variables for which data were sought (e.g. participant and intervention characteristics, funding sources). Describe any assumptions made about any missing or unclear information. | Section 2.4 "Data Extraction": study characteristics extracted (first author, year, subtype, drug, experimental system, resistance model type, ferroptosis targets, intervention strategy, comparator groups) |
| Study risk of bias assessment | 11 | Specify the methods used to assess risk of bias in the included studies, including details of the tool(s) used, how many reviewers assessed each study and whether they worked independently, and if applicable, details of automation tools used in the process. | Section 2.5 "Methodological Quality Assessment": SYRCLE tool for in vivo studies (8 domains, 0‑8 scoring); adapted OHAT framework for in vitro studies (7 domains, 0‑7 scoring); two independent reviewers; results in Supplementary Tables S3‑S4 |
| Effect measures | 12 | Specify for each outcome the effect measure(s) (e.g. risk ratio, mean difference) used in the synthesis or presentation of results. | Section 2.4 "Data Extraction": outcomes extracted as quantitative measures (IC50 values, cell viability percentages, tumor volume, etc.); narrative synthesis precluded meta-analysis |
| Synthesis methods | 13a | Describe the processes used to decide which studies were eligible for each synthesis (e.g. tabulating the study intervention characteristics and comparing against the planned groups for each synthesis (item #5)). | Section 2.6 "Data Synthesis": studies grouped according to chemotherapeutic class and mechanistic categories; comparative analysis focused on consistency across models |
|  | 13b | Describe any methods required to prepare the data for presentation or synthesis, such as handling of missing summary statistics, or data conversions. | Section 2.4 "Data Extraction": data extracted directly from published text, tables, and figures; authors not contacted for missing data |
|  | 13c | Describe any methods used to tabulate or visually display results of individual studies and syntheses. | Section 3 "Results": Tables 1‑5 and Figures 1‑3 present summarized data; Supplementary Tables S1‑S4 provide detailed study‑level data |
|  | 13d | Describe any methods used to synthesize results and provide a rationale for the choice(s). If meta-analysis was performed, describe the model(s), method(s) to identify the presence and extent of statistical heterogeneity, and software package(s) used. | Section 2.6 "Data Synthesis": narrative synthesis chosen due to substantial heterogeneity (study design, resistance models, ferroptosis assays, outcomes); meta-analysis not performed |
|  | 13e | Describe any methods used to explore possible causes of heterogeneity among study results (e.g. subgroup analysis, meta-regression). | Section 3.4 "Cross‑analysis of Mechanisms and Drug Classes": subgroup analyses by drug class and mechanism |
|  | 13f | Describe any sensitivity analyses conducted to assess robustness of the synthesized results. | Not applicable (narrative synthesis, no meta-analysis). |
| Reporting bias assessment | 14 | Describe any methods used to assess risk of bias due to missing results in a synthesis (arising from reporting biases). | Section 2.5 "Methodological Quality Assessment": SYRCLE and OHAT tools include domains for selective outcome reporting and outcome consistency |
| Certainty assessment | 15 | Describe any methods used to assess certainty (or confidence) in the body of evidence for an outcome. | Section 3.5 "Evidence Strength Ranking of Ferroptosis Mechanisms": developed a ranking system (Levels A‑C) based on number of studies, in vivo validation proportion, and drug class coverage |
| **RESULTS** | | |  |
| Study selection | 16a | Describe the results of the search and selection process, from the number of records identified in the search to the number of studies included in the review, ideally using a flow diagram. | Section 2.1 "Search Strategy" and Figure 1 (PRISMA flow diagram): 44 studies included from 467 records after deduplication, full‑text screening, and exclusion |
|  | 16b | Cite studies that might appear to meet the inclusion criteria, but which were excluded, and explain why they were excluded. | Section 2.1 "Search Strategy": 15 excluded |
| Study characteristics | 17 | Cite each included study and present its characteristics. | Section 3.6 "Efficacy of Ferroptosis‑Mediated Chemosensitization": all 44 studies cited with key findings; Table 1 and Supplementary Tables S1‑S2 provide complete characteristics |
| Risk of bias in studies | 18 | Present assessments of risk of bias for each included study. | Section 3.2 "Risk of Bias and Methodological Quality Assessment": summarizes SYRCLE and OHAT results; Supplementary Tables S3‑S4 provide domain‑by‑domain scoring |
| Results of individual studies | 19 | For all outcomes, present, for each study: (a) summary statistics for each group (where appropriate) and (b) an effect estimate and its precision (e.g. confidence/credible interval), ideally using structured tables or plots. | Section 3.6 summarizes key findings; Table 5 presents quantitative synthesis (rescue %, in vivo %); Supplementary Table S2 provides detailed outcomes per study |
| Results of syntheses | 20a | For each synthesis, briefly summarise the characteristics and risk of bias among contributing studies. | Sections 3.3-3.5: Each mechanistic category is described with associated studies; risk of bias context is provided in Section 3.2. |
|  | 20b | Present results of all statistical syntheses conducted. If meta-analysis was done, present for each the summary estimate and its precision (e.g. confidence/credible interval) and measures of statistical heterogeneity. If comparing groups, describe the direction of the effect. | Section 3.3-3.6: Narrative synthesis with descriptive statistics (counts, percentages); no meta-analysis performed. |
|  | 20c | Present results of all investigations of possible causes of heterogeneity among study results. | Section 3.4 "Cross‑analysis of Mechanisms and Drug Classes": explores heterogeneity by drug class and mechanism |
|  | 20d | Present results of all sensitivity analyses conducted to assess the robustness of the synthesized results. | Not applicable (narrative synthesis, no meta-analysis). |
| Reporting biases | 21 | Present assessments of risk of bias due to missing results (arising from reporting biases) for each synthesis assessed. | Section 3.2: SYRCLE and OHAT assessments include selective outcome reporting; all 44 studies demonstrated good outcome consistency |
| Certainty of evidence | 22 | Present assessments of certainty (or confidence) in the body of evidence for each outcome assessed. | Section 3.5 "Evidence Strength Ranking of Ferroptosis Mechanisms" and Table 4: provides evidence strength levels (A‑C) for each mechanism |
| **DISCUSSION** | | |  |
| Discussion | 23a | Provide a general interpretation of the results in the context of other evidence. | Section 4.3 "Comparison with Prior Research" |
|  | 23b | Discuss any limitations of the evidence included in the review. | Section 4.5 "Limitations of the Evidence" |
|  | 23c | Discuss any limitations of the review processes used. | Section 4.5 "Limitations of the Evidence": Includes discussion of CDX model limitations, subtype underrepresentation, absence of standardized outcome measures, and reporting deficiencies. |
|  | 23d | Discuss implications of the results for practice, policy, and future research. | Section 4.4 "Clinical Implications and Translational Potential" and Section 4.6 "Future Directions" |
| **OTHER INFORMATION** | | |  |
| Registration and protocol | 24a | Provide registration information for the review, including register name and registration number, or state that the review was not registered. | Title page: "Systematic Review Registration: PROSPERO CRD420261334827" |
|  | 24b | Indicate where the review protocol can be accessed, or state that a protocol was not prepared. | Title page: "The review protocol is available at: https://www.crd.york.ac.uk/PROSPERO/view/CRD420261334827" |
|  | 24c | Describe and explain any amendments to information provided at registration or in the protocol. | PROSPERO amendment: search expanded to include Embase and Web of Science; search date extended to May 26, 2026; number of included studies updated from 29 to 44 |
| Support | 25 | Describe sources of financial or non-financial support for the review, and the role of the funders or sponsors in the review. | Funding section: "This research received no external funding." |
| Competing interests | 26 | Declare any competing interests of review authors. | Competing Interests section: "The authors declare that there are no competing interests associated with the manuscript." |
| Availability of data, code and other materials | 27 | Report which of the following are publicly available and where they can be found: template data collection forms; data extracted from included studies; data used for all analyses; analytic code; any other materials used in the review. | Data availability statement (page 24): "Not applicable."; Supplementary Tables S1‑S4 include all extracted data; Supplementary File 1 includes complete search strategies |

*From:*  Page MJ, McKenzie JE, Bossuyt PM, Boutron I, Hoffmann TC, Mulrow CD, et al. The PRISMA 2020 statement: an updated guideline for reporting systematic reviews. BMJ 2021;372:n71. doi: 10.1136/bmj.n71. This work is licensed under CC BY 4.0. To view a copy of this license, visit <https://creativecommons.org/licenses/by/4.0/>
